# Efficacy of Kinesiotape to Improve Upper-Extremity Function in Children and Adolescents with Cerebral Palsy: A Systematic Review

**DOI:** 10.3390/children11040480

**Published:** 2024-04-17

**Authors:** Victoria Calvo-Fuente, Concepción Soto-Vidal, Ana Ramón-Corcoba, Ester Cerezo-Téllez, Yolanda Pérez-Martín, Soraya Pacheco-da-Costa

**Affiliations:** 1 Neuromusculoskeletal Physical Therapy in Stages of Life Research Group (FINEMEV), Department of Nursing and Physical Therapy, Faculty of Medicine and Health Sciences, Universidad de Alcalá, Autovía A2, km 33.200, Alcalá de Henares, 28805 Madrid, Spain; victoria.calvo@uah.es (V.C.-F.); ester.cerezo@uah.es (E.C.-T.); soraya.pacheco@uah.es (S.P.-d.-C.); 2 Centro Angel Vacas, Torres de la Alameda, 28813 Madrid, Spain; a.ramon@edu.uah.es; 3 Humanization in the Intervention of Physiotherapy for the Integral Attention to the People (HIPATIA), Department of Nursing and Physical Therapy, Faculty of Medicine and Health Sciences, Universidad de Alcalá, Autovía A2, km 33.200, Alcalá de Henares, 28805 Madrid, Spain; yolanda.perez@uah.es

**Keywords:** cerebral palsy, kinesiotape, upper-extremity function, children, adolescent, physical therapy

## Abstract

Background: Cerebral palsy (CP) is one of the primary causes of physical disabilities in children that affects posture and movement. Upper-extremity (UE) function is frequently impaired, which may result in activity and participation limitations in people with CP. The use of kinesiotape (KT) has increased in the treatment of CP for various purposes. The aim of this systematic review was to assess the efficacy of KT for improving UE function in children and adolescents with CP. Methods: The literature search was carried out in PubMed, Cochrane, PEDro, Web of Science and SCOPUS databases. The methodological quality was analyzed with the PEDro scale. Review Manager (RevMan 5.4.1) was used for data extraction and risk of bias assessment. Results: A total of five randomized clinical trials were included. The use of KT showed improvement in UE functionality in three studies, with significant outcomes for range of motion (ROM) (three studies), fine motor skills (two studies), grip strength (one study) and manual dexterity (one study). Moreover, it also showed significant improvements in spasticity and gross motor function (one study). Overall, methodological quality was moderate, and the risk of bias was high in the domains related to blinding. Conclusion: The use of KT showed improvement in UE function in children and adolescents with CP. However, further research is needed to reinforce the conclusions on the efficacy of KT as a therapeutic tool.

## 1. Introduction

Cerebral palsy (CP) refers to a heterogeneous group of conditions involving permanent motor dysfunction which affects muscle tone, posture and movement. It is caused by damage or abnormalities in the development of the brain. This fact limits the brain’s ability to control movement and maintain posture and balance, which may have a negative impact on functionality and participation [1].

One of the main goals in physical therapy (PT) interventions for children and adolescents with CP is to promote the highest level of autonomy in routines and functioning for activities of daily living (ADL), especially related to bimanual function [2]. There is a correlation between manual dexterity, described in the Manual Ability Classification System (MACS), and general mobility, locomotion, communication, socialization and personal care [3]. Impairments in the upper extremity (UE) are a major factor for activity limitation and participation restriction in individuals with CP and may affect up to 50% of CP subjects [4,5]. UE limitations are mostly due to a lack of trunk control, decrease in shoulder girdle motor control and imbalance between spastic and paretic muscles [6]. That makes it difficult for people with CP to perform UE-specific tasks, such as reaching, grasping and manipulation [4], and it leads to the significant involvement of the positioning and functioning of the elbows, wrists and hands [7]. Moreover, a lack of autonomy and dependence on other people may affect the individuals’ quality of life [4,5,6].

Kinesiotape (KT) is a type of elastic, cotton, adhesive bandage that simulates the flexibility and stretchiness of human skin, muscle and fascia. It is one of the PT techniques that are currently used for the treatment of subjects with CP [8,9]. It is usually used in combination with other PT techniques because it is easy to use, inexpensive and can be removed or adjusted depending on the intervention goals [10].

In recent years, it has increasingly been used in people with different neurological disorders in order to correct postural alignment, increase joint stability [11], activate weak musculature, control spasticity [12], stimulate mechanoreceptors through skin stretching during movement to increase sensation and proprioception [7], engage motor unit recruitment [13], improve voluntary movement control and coordination [14,15] and relieve pain [16].

KT applied to children with CP has shown beneficial effects on gross and fine motor function [7,8,17,18], functionality [8,19] and postural control and stability [8]. In addition to that, wearing the bandage may encourage children to perform the actions during which there are the most limitations [8]. It has also been shown to be effective in mild-to-moderate CP [8,18,20] and better outcomes are reached when combined with other PT techniques such as neurodevelopmental treatment, neuromuscular electrical stimulation or therapeutic exercises [8,20,21].

Recent systematic reviews and meta-analyses have evaluated the use of KT in different ways. Some studies investigated early therapeutic approaches for the UE in hemiplegic CP [7], others assessed the effectiveness of KT in different PT interventions either for children with CP [8,9,10,18,22] or for people with different neurological conditions [19], and some assessed the effects of KT on motor function in children with motor impairments [23]. Nevertheless, currently there is a lack of comprehensive review examining KT effects specifically on UE in children and adolescents with CP. 

Therefore, the aim of this systematic review was to assess the efficacy of KT for improving UP function in children and adolescents with CP.

## 2. Materials and Methods

The systematic review followed the Preferred Reporting Items for Systematic Reviews and Meta-Analyses (PRISMA) Guidelines (see Supplementary Materials). It is registered in PROSPERO—International prospective register of systematic reviews website with registration number CRD42023469473.

### 2.1. Search Strategy

The literature search was carried out during March 2023, with no date limit, in PubMed, Cochrane, PEDro, Web of Science and SCOPUS databases. The search strategy for all databases was performed using descriptors and combinations of terms referring to cerebral palsy and kinesiotape, such as “Kinesiotape”, “Kinesio tape”, “Kinesiotaping”, “Athletic tape”, “Neuromuscular bandage”, “Neuromuscular bandaging”, “Neuromuscular tape”, “Cerebral Palsy”, “Cerebral Palsy, Spastic, Diplegic” and “Cerebral Palsy, Spastic Quadriplegic”. Manual search was conducted for references in the selected articles to identify additional relevant studies.

### 2.2. Eligibility Criteria

The selection process was performed according to the PICOS framework. Randomized clinical trials, without time restrictions, and published in Spanish or English were included if they met the following criteria: Population—studies involving only children and adolescents aged 3–18 years diagnosed with CP; Interventions—KT use on UE combined or not with other PT interventions; Comparator—KT intervention group with a control group; Outcomes—focused on UE, such as functionality, fine motor skills, grip strength, range of motion (ROM), spasticity, manual dexterity and gross motor function (GMF).

Other types of publications such as letters to editor, conference abstracts, pilot studies and protocols, reviews or meta-analyses were excluded.

### 2.3. Study Selection

The retrieved papers were imported to the Covidence platform of The Cochrane Collaboration [24] to remove duplicate results and perform peer review. An initial selection of the studies that met the selection criteria was performed based on the information available in the title and abstract. A second selection phase was performed and the studies’ full texts were analyzed. The studies were selected if they met all the inclusion criteria.

### 2.4. Data Extraction

The data were extracted from the selected studies using The Cochrane Collaboration’s Review Manager 5.4.1 software [25] and information on participant characteristics, interventions, variables and outcomes was collected.

### 2.5. Assessment of Methodological Quality and Risk of Bias

Physiotherapy Evidence Database (PEDro) Scale was used to assess the methodological quality of the studies. This scale consists of 11 items related to the validity of the articles assessed. The first criterion is related to external validity; however, it is not scored. The remaining 10 items relate to internal validity. These criteria are used to assess issues related to randomization and allocation blinding; homogeneity of groups; blinding of participants, therapists and assessors; and outcome measures [26]. The scores ranged from 0 to 10, where a score below 3 would correspond to low methodological quality, 4 to 5 to moderate quality, 6 to 8 to high quality and 9 to 10 to excellent quality [27].

Review Manager 5.4.1 software [25] was used to assess the risk of bias. This assesses seven domains: random sequence generation (selection bias), allocation concealment (selection bias), blinding of participants and personnel (performance bias), blinding of outcome assessment (detection bias), incomplete outcome data (attrition bias), selective reporting (reporting bias) and other biases. Each domain was classified as low risk of bias, high risk of bias or unclear risk of bias when not expressed in the study.

All search, selection, data extraction and quality assessment processes were performed by two independent reviewers (A.R.C. and V.C.F.). Disagreements were resolved by a third reviewer (S.P.d.-C.).

## 3. Results

### 3.1. Study Selection

**Figure 1:**
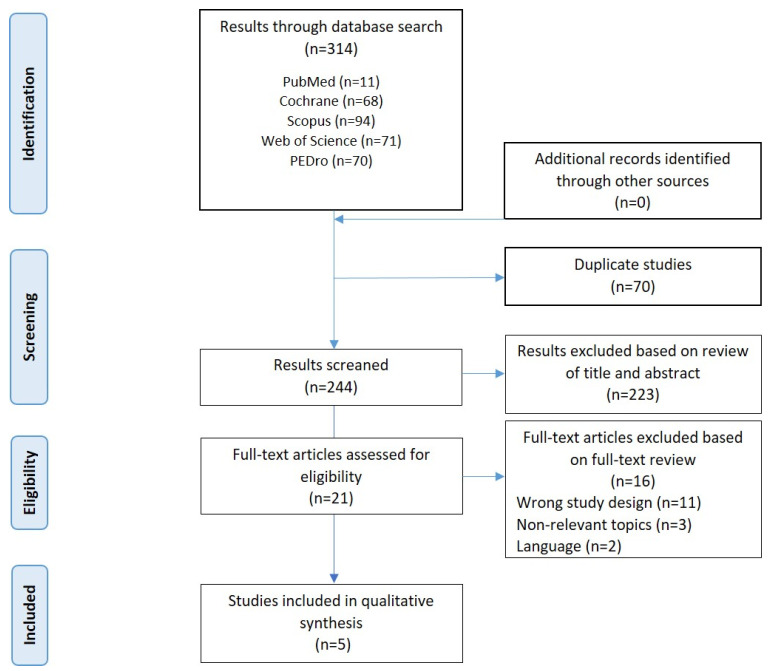
Flowchart of the selection process.

A total of 314 studies were retrieved from the five databases searched. A total of 70 duplicated studies were discarded, leaving 244 studies for peer review. After reading the titles and the abstracts of the potentially relevant articles, 223 articles were excluded. Of the 21 studies selected to assess their eligibility for a full-text reading, 11 studies were discarded due to not accomplishing the criteria of study design (pilot studies, posters, conference abstracts, clinical trial registries), 3 studies were discarded because the aims were irrelevant for the review and 2 studies were discarded because they were written in a language other than Spanish or English. Therefore, 16 studies were excluded, and the final sample was of five articles [28,29,30,31,32]. The selection process is shown in the flowchart seen in Figure 1. 

### 3.2. Synthesis of Results and Studies’ Characteristics

The characteristics of the included studies are available in Table 1. They are ordered by publication date from less recent to most recent.

#### 3.2.1. Participants

The final sample of this qualitative synthesis included 173 participants after two drop-outs (1.15%). The studies included samples of children and adolescents diagnosed with CP at ages between 3 and 18 years old. The sample of three studies [28,29,32] included 97 children with hemiparesia, 21 quadriparietic, four diparetic and one triparetic subjects; a study [30] did not provide this information; another study [31] indicated bilateral involvement in 14 subjects, right upper extremity (RUE) in six subjects, and left upper extremity (LUE) in ten subjects.

#### 3.2.2. Interventions

Four studies [28,29,31,32] applied KT to the hand, wrist or forearm of the affected UE, and one study [30] applied it to the shoulder. Three studies [28,29,30] described KT as the unique intervention, while the other two studies [31,32] combined it with other PT techniques, such as neuromuscular electrical stimulation (NES) for wrist extensors [31]; and wrist wheel (WW) on forearm supination ROM [32]. Subjects in CG received no intervention [29,30] or received a placebo [28]. The number of sessions were from 1–2 [28,29,30] to 20–36 sessions [31,32], and they included pre- and post-intervention assessment [28,29,30,31], short-term (two-day) post-intervention follow-up [28] and 3 months post-intervention follow-up [32]. The session duration was in the range of 20–45 min. Some studies included only one session with pre- and post-intervention assessments [29,30].

#### 3.2.3. Outcomes Measures

ROM was measured in three studies [28,31,32] with goniometry in two of them and by a computer program using the Kinect V2 sensor in the other; two studies [29,31] assessed fine motor skills, one using the “Nine Parts Puzzle Test” and “Nine Hole Peg Test”, and the other using the Minnesota Hand Skill Test. Three studies [30,31,32] measured UE functionality, one using the Duruoz Hand Index and the Jebsen Hand Function Test, one of them using virtual reality software and the Box and Block Test (BBT) and the other using the SHUEE Shriners Hospital Upper Extremity Evaluation (SHUEE) divided into two sections: spontaneous functional analysis (SFA) and dynamic positional analysis (DPA). One study [28] measured grip strength using vigorimetry, and in another study [31] spasticity was assessed with the modified Ashworth Scale (MAS), GMF using the gross motor function classification system (GMFCS) and manual dexterity with MACS. Quantitative synthesis was not feasible due to the great heterogeneity of the outcome measures.

### 3.3. Methodological Quality and Risk of Bias

The PEDro scale was used to determine the methodological quality of the studies. Two studies [28,29] obtained a moderate methodological quality score (5/10) and three of them [30,31,32] had a high methodological quality score (6–7/10), as shown in Table 2. The mean methodological quality score was 5.8 points; therefore, this systematic review obtained a moderate methodological quality score overall. All the included studies [28,29,30,31,32] had a random allocation, provided an adequate follow-up, described between-group comparisons and point estimates and variability. The criteria related to subjects, therapists and blind assessors were not described or were unclear. 

**Figure 2:**
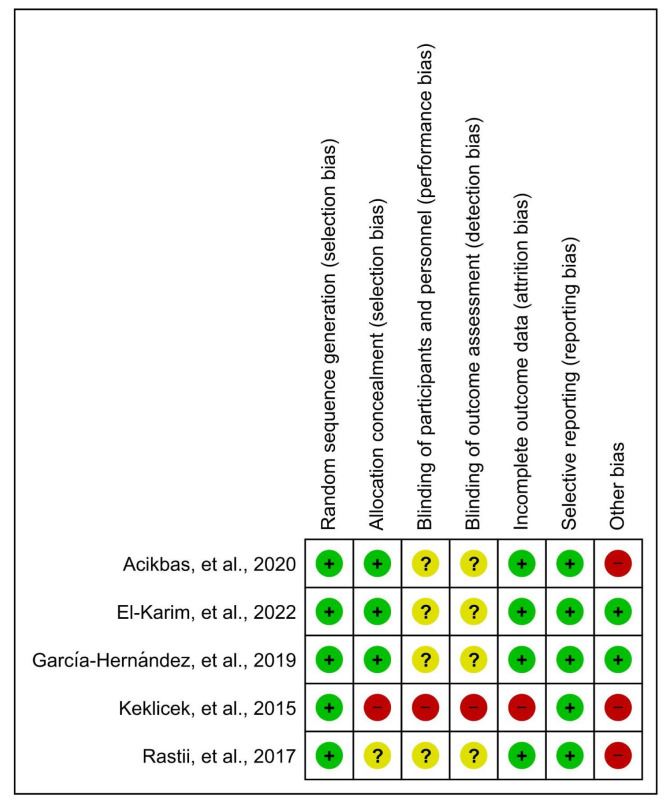
Risk of bias of the included studies [28,29,30,31,32].

Review Manager (RevMan 5.4.1) was used in all studies. All of them [28,29,30,31,32] showed a low risk for random sequence generation. In the allocation concealment, one study showed high risk [29], one study unclear risk [28] and three studies showed low risk [30,31,32]. Regarding the performance and detection bias, one study showed high risk [29] and the other four studies unclear risk [28,30,31,32]. Regarding participants and personnel blinding, one study showed high risk [29] and four studies an unclear risk [28,30,31,32]. Related to attrition bias, four studies showed low risk [28,30,31,32] with one study showing high risk [29]. All studies showed low risk for reporting bias [28,29,30,31,32]. Other biases with high risks were those related to the lack of homogeneity in the sample at the beginning [28,29] and to the subjective evaluation of some parameters [31], while studies [30,31,32] showed low risk (Figure 2). 

### 3.4. Efficacy of Interventions and Adverse Effects

In terms of ROM, there were significant changes intergroup for EG with KT for three studies [28,31,32]. Regarding intergroup comparison, in one study [28] there were significant differences between groups in favor of the EG (KT applied with tension) for wrist extension and thumb extension and abduction after KT application, 2 days after intervention and 2 days after KT removal. One study [31] showed significant differences in elbow flexion in the KT group; and in shoulder flexion or abduction in the NES group. Notably, the NES group showed more favorable changes than the KT group. The study [32] showed better results in forearm ROM supination in the WW group than in the KT group.

Regarding UE functionality, there were relevant changes in EG with KT in three studies [30,31,32]. In one study [30], favorable changes were obtained in the post-test in EG (KT on shoulder) in the intragroup analysis in terms of speed of action, energy expenditure, smoothness of movement and stability of movement. There were no significant changes in accuracy or movement trajectory. Study [31] showed no differences in functionality in the intergroup results (KT and NES). In study [32], the WW group showed better results in the DPA than the KT group.

Studies [29,30,31] found significant changes in fine motor skills in group comparisons of EG with KT in the pre- and post-KT measurements. In the intergroup comparison, study [29] obtained significant differences in favor of the intervention KT group, while study [31] showed significant differences in favor of the NES-treated group. 

Grip strength was assessed by a study [28] wherein significant differences were obtained between the control and intervention groups (KT with tension) in favor of the intervention group, and beneficial effects were observed after application, 2 days after application and 2 days after removal.

Spasticity showed significant improvements in wrist flexion and forearm supination in both groups (KT and NES) in one study [31]. Regarding GMF, in one study [31], two of the participants in the KT group improved one level on the GMFCS, while in the NES group one participant improved one level on this scale. In the study [31], six participants in the KT group improved by one level on the MACS, and three participants in the NES group improved by one level. 

None of the studies reported adverse effects.

## 4. Discussion

The purpose of this systematic review was to determinate the efficacy of the KT application for improving UE function in children and adolescents with CP, through the analysis of randomized clinical trials published to date. 

The number of participants included in the trials was 173, which may reduce the precision of the findings, although the sample size is usually small in this type of studies. Most of the participants were children and adolescents diagnosed with hemiparesis, followed by quadriparesis, diparesis and triparesis. This could be justified because hemiparesis is the type of CP wherein the UE is usually most affected [33,34,35]. The participants’ wide age range included in the studies (3–18 years old) may represent a drawback due to developmental differences; however, there are several current systematic reviews on CP interventions that use similar age ranges [36,37,38,39]. 

Regarding the intervention, KT was used either as a unique technique or in combination with other PT techniques. Prior studies showed that the use of KT in combination with other PT techniques is more effective for improving GMF and autonomy in performing ADL [8,9,18,22,23].

The application techniques of KT in all cases proved to be different and were compared with a CG or with another EG. For this reason, there is no evidence of any KT technique being more effective than another as each study used them for different purposes. There was a great variety regarding number and duration of sessions. The most homogeneity in the studies analyzed was in terms of pre- and post-treatment assessment, as well as the short-term follow-up, while long-term outcomes may be more interesting [7].

Despite KT techniques’ variety, the evidence for many of them is still unclear. Further research and well-established protocols are needed to generate evidence regarding settings, duration of interventions and sessions and modalities of KT use [10,19,23]. Four studies [28,29,31,32] applied KT to the hand, wrist or forearm of the affected UE. Only one study [30] applied KT on the shoulder to stimulate deltoid muscle function, by providing mechanical correction, stability and facilitating UE movement. Two studies [28,31] placed it on the dorsal area of the wrist and fingers, to improve the function of the wrist and finger extensor muscles as well as the thumb abductor and extensor; another study [29] inhibited the thumb in the palm of the hand by placing KT on the extensor surface of the thumb; while in another one [32] KT was placed on the volar aspect of the forearm to improve forearm supination. Wrist and hand involvement is often significant in CP, and improving the position of both could encourage the child to use the affected limb more and reduce the occurrence of secondary musculoskeletal problems [7]. 

The studies did not measure the same outcomes or used the same instruments for outcome measurement. The outcomes variables were ROM [28,31,32], UE functionality [30,31,32], fine motor skills [29,31], grip strength [28], spasticity, GMF and manual dexterity [31]. Quantitative synthesis was not feasible, due to the great heterogeneity of the outcomes measured in the studies.

There was a significant improvement in ROM in three studies when KT was applied to the wrist [28,31,32] thumb [28], elbow and forearm [31,32]. However, in the study [32], WW exercises were more effective for supination ROM improvement. These results are probably due to the fact that WW improves proprioception considering visual feedback which allows the ROM to increase, by setting up stimuli for the child to perform supination exercises [32]. The positive effects of KT were demonstrated in previous studies. After 45 min of KT on the wrist extensor muscles in children with CP, there were statistically significant differences in wrist extension and lateral deviations. KT can correct abnormal hand posture, bring the hand into a functional position by stimulating the extensor muscles and inhibiting the flexor muscles of the wrist, thus improving ROM [40]. Several authors concluded that KT provides joint support, stimulates cutaneous mechanoreceptors, increasing proprioceptive inputs to muscles and enhancing their recruitment or contributing to the inhibition of muscle tone [7,17,29], thus allowing for optimal functional movement [8,23].

Spasticity was assessed in one study [31] which found good results in both the KT and NES groups. This may be due to the stimulation of cutaneous receptors in the antispasticity position by KT, together with the administration of stimulation that activates contraction and improves muscle strength. However, the effect of KT and NES on spasticity reduction is unclear as they were combined with neurodevelopmental treatment (NDT). In the study by Toxqui et al. [41], a significant decrease in spasticity was observed when KT was applied to the trunk. Other studies assessing the effects of KT on spasticity in stroke patients also had good results in reducing spasticity [42,43,44]. There is a hypothesis according to which KT used for a long time promotes muscle stretching and could cause the autogenic inhibition of hypertonic muscles [44].

Studies assessing fine and gross motor skills [29,31] showed good results in the groups wherein KT was applied to the hand, wrist and forearm, although the study [31] showed better results in the NES group. Treatment with NES appears to be effective on UE function and performance in CP because it improves muscle strength and reduces spasticity. Chitaria et al. [45] also obtained good short-term results in fine motor skills by applying KT to the wrist extensors for 3 days in subjects with CP. Hoşbaş et al. [46] applied KT on wrist and finger extensors in children with unilateral spastic CP, obtaining good post-intervention results in fine motor skills and a significant improvement in gross motor skills of the KT group compared to the vibration therapy and control group. The literature review showed that KT can be effective as part of the PT intervention to improve GMF and dynamic activities, especially at higher motor and developmental stages in people with CP [8,9,18,20,22,23].

Mobility and grip strength are limited by the deformity and abnormal posture of CP. Rastii et al. [28] measured grip strength by vigorimetry and obtained positive results that were maintained two days after KT application and two days after its removal. Lemos et al. [47] showed that KT increased the handgrip strength of 75 healthy women, and it was maintained for 48 h. Mohamed et al. [33] found that mirror therapy combined with KT had a positive effect for improving UE function quality, dexterity and grip strength in children with hemiplegic CP. However, Elham et al. [48] investigated the effects of KT on grip and release functions in children with spastic hemiparesis CP and did not obtain significant changes in comparison with pre-treatment. The difference in results in the study could be due to the measurement instruments used (motor and quality of hand skills tests and scales) instead of the vigorimeter which may help to better detect small changes in hand functions. Furthermore, the study only included eleven CP children, a small sample which reduces the power of the results.

Beneficial effects of KT for UE functionality were found in two studies [30,31]. The study [31] showed good results in both treatment groups (KT and NES); however, there were no differences in intergroup comparison. This result is probably due to the fact that both groups received additional NDT. The study [30] obtained good results regarding speed of action, energy expenditure, smoothness of movement and stability of movement, and the results of clinical measures (Box and Block Test scores) revealed that KT has an immediate impact on UE function. Several studies agree that the use of KT improves UE function in children with CP [49,50], increasing their functional independence and helping them to develop the demands of ADL [10,23,51]. However, there are discrepancies regarding the immediate effect on UE functional abilities [50] and this should be studied further in future research [10].

Manual dexterity to manipulate objects was analyzed in the study [45], showing beneficial effects by increasing one level on the MACS in both treatment groups (six children in the KT group and three children in the NES group). The application of KT combined with NDT had a positive effect on the ROM and spasticity of hand and wrist muscles, facilitating object manipulation. 

Among the strengths of the study, as far as the authors know, it is the first systematic review on the efficacy of KT to improve UE function in children with CP. The literature search was conducted with no date limits in order to be more exhaustive. And the inclusion of randomized clinical trials allowed for reaching the highest degree of evidence. Throughout the process, studies were assessed independently by two reviewers to reduce the risk of bias. In case of disagreement, a third reviewer was consulted to reach a final decision. 

However, there are certain limitations to this study. First of all, potentially relevant studies may not have been identified through the search strategy used, or in the databases consulted. On the other hand, the sample size of five studies which were included in this analysis may be considered small. In addition, the included studies showed heterogeneous aspects in terms of age and type of CP, as well as the types of interventions, which limits the reliability and generalizability of the findings. The quality of the systematic review is affected by the quality of the included studies, in which the methodological quality was moderate and the risk of bias was high or unclear, especially in the domains related to blinding of the participants and researchers, due to the difficulty of applying blinded techniques in PT interventions. The heterogeneity in terms of aims, methodology and interventions may result in difficult qualitative synthesis and may introduce possible confounding variables that could influence the results. Therefore, it is important to interpret these results with caution. 

## 5. Conclusions

The results of this systematic review suggest that the use of KT has beneficial effects on UE function, including ROM, gross and fine motor function, grip strength, spasticity and manual dexterity, in both children and adolescents with CP. However, further research in needed, with more specific KT procedure descriptions, studies of higher methodological quality, with larger and more homogeneous samples and long-term follow-up in order to reinforce the conclusions on the efficacy of KT as a therapeutic tool.

## Figures and Tables

**Table 1:** Studies’ characteristics (*n* = 5).

Study (Year)	Participants	Objective	Intervention	Variables	Results
Keklicek et al., 2015 [29]	N = 45 Children aged 4–14 years with CP. Spasticity 2–3 (MAS) in hand or wrist, without ROM limitation UE. CG = 15 (13 hemiparesis, 2 quadriparesis) TG = 15 (13 hemiparesis, 2 quadriparesis) TPPG = 15 (12 hemiparesis, 2 quadriparesis,1 triparesis).	To prove the effects of the application of thenar palmar KT, with and without pressure, on UE function in children with CP.	CG: No intervention. TG: KT on thumb extensors and 3 strips on the wrist and first interdigital space to avoid thumb opposition. TPPG: KT on thumb extensors + polyurethane piece to press on the thenar eminence. Duration of treatment: 1 session of 20 min. Evaluation before and after treatment.	Fine motor skills: -The Nine Hole Peg Test. -Nine Parts Puzzle Test.	Fine motor skills: Intragroup: CG unchanged. TG and TPPG: statistically significant differences after 20 min of KT application. Intergroup: Differences between CG and TG and TPPG in favor of the last ones showed that KT with or without pressure was effective in improving fine motor skills. There were no differences between TG and TPPG. TPPG maintained positive effects after 20 min of KT removal. There was no additional effect from the use of pressure with bandaging (*p* = 0.22).
Rastii et al., 2017 [28]	N = 32/30 Children aged 4 to 14 with CP. Spasticity less than 3 (MAS) in hand or wrist, without UE ROM limitation. EG = 17/15 (5 hemiparesis, 3 diparesis, 7 quadriparesis). RUE affected: 11, LUP affected: 4 CG = 15 (6 hemiparesis, 1 diparesis, 8 quadriparesis). Affected, RUE: 11, LUP: 4	To investigate the effectiveness of KT on hand active ROM and hand grip in children with CP.	EG: KT in wrist extensors and thumb abductor. A 30% tension was applied on muscles and 75% on joints. The purpose was to improve muscle function and correct wrist flexion and thumb opposition. CG: same application of KT without tension. Duration of treatment and follow-up: assessment before and immediately after KT application; 2 days after application and 2 days after removal.	ROM: -Goniometry Grip strength: -Vigorimetry	ROM: Significant differences between groups (*p* < 0.05) in favor of EG in wrist extension and thumb extension and abduction, after placing the KT; 2 days later; and 2 days after KT removal only in wrist extension. Grip strength: Significant differences in favor of EG after KT application in all assessments.
García-Hernández et al., 2019 [30]	N = 20 Children aged 6 to 13 years with CP. Mild motor impairment (GMFCS I-II) and cognitive function. EG = 10 CG = 10	To evaluate the effects of KT in arm motion in function, task performance, kinematic and dynamic cost functions.	EG: KT testing on affected shoulder. Three strips: one in “Y” to correct the shoulder joint (50% tension); one in “Y” to stimulate the deltoid (15% tension); and one in “I” to stimulate shoulder abduction (50% tension). CG: tests without KT. Duration of treatment: 1 session. Pre- and post-test evaluation.	Functionality: -BBT: UE functionality. -Virtual reality: reaching for objects (reaching for 3 balls and putting them in a box). -Performance was measured: speed and accuracy, hand trajectory, ROM, smoothness and stability of movement and joint energy expenditure.	Functionality: Only the EG obtained significant changes in BBT (*p* < 0.01). Virtual reality: only the EG had favorable changes in the post-test in shoulder flex reduction (*p* < 0.01), speed of action (*p* < 0.05), energy expenditure (*p* < 0.01) and movement smoothness and stability. No differences between groups in level of accuracy achieved and trajectory. More time to complete the reaching phase than the carrying phase was needed in both groups.
Acikbas et al., 2020 [31]	N = 30 Children from 3 to 18 years old with CP. Ability to adapt to the exercises. NESG = 15 KTG = 15 RUE affected: 6 LUE: 10, Bilateral: 14.	To estimate the effect of the KT and NES addition to NDT on ROM, muscle tone and UE function in children with CP.	NESG: NES on wrist extensors + NDT. Treatment session: 15 min of NES + 30 min of NDT. KTG: KT in wrist extensors + NDT. Treatment session: KT applied for 2 days + 30 min of NDT. Duration of treatment: 2 days/week, for 10 weeks. Pre and post-treatment evaluation.	Hand functionality: -Duruoz Hand Index -Jebsen Hand Function Test Fine motor skills: -Minnesota Hand Skill Test ROM: -Goniometry Spasticity: -MAS Gross motor function: -GMFCS Manual dexterity: -MACS	Hand functionality: there were statistically significant changes in both groups. No significant differences between groups (*p* > 0.05). Fine motor skills: Intragroup: significant changes in both (*p* = 0.001). Intergroup: significant difference in favor of NESG (*p* = 0.02). ROM: Intragroup: statistically significant changes in KTG for shoulder abduction, elbow extension, supination, wrist flex and wrist extension; In NESG there were statistically significant changes in shoulder flex and abduction, supination, wrist flexion and extension. Intergroup: there were differences in elbow flex in favor of KTG (*p* = 0.035), and in shoulder flexion (*p* = 0.000) and abduction (*p* = 0.001) in NESG. Spasticity: both groups showed significant changes in wrist flexion and supination. No significant differences between groups. Gross motor function: in KTG 2 participants improved one level in GMFCS; in NESG 1 participant improved one level in GMFCS. Manual dexterity: in KTG 6 participants improved one level in the MACS; in NESG 3 participants improved one level in the MACS.
El-Karim et al., 2022 [32]	N = 48 Children aged 6 to 8 years old with UCP. Spasticity 1 and 1+ (MAS) in UE. Functional impairment of the hand level II and III in MACS. EG = 24 CG = 24	To compare between KT and WW effect on forearm supination ROM and its reflection on functions of UL in children with UCP.	KTG: KT on forearm + PT. WWG: WW + PT. Duration of treatment: 3 days/week, for 3 months. Evaluation before and after group interventions.	Supination ROM: -Goniometry UE Functionality: -SHUEE	Intragroup: statistically significant changes in both groups (*p* < 0.001). Intergroup: statistically significant differences in favor of WWG (*p* < 0.001).

Abbreviations. CP: cerebral palsy; MAS: modified Ashworth scale; ROM: range of motion; UE: upper extremity; CG: control group; TG: taping group; TPPG: taping plus palmar; KT: kinesiotape; EG: experimental group; RUE: right upper extremity; LUP: left upper extremity; GMFCS: gross motor function classification system; BBT: box and block test; NESG: neuromuscular electrical stimulation group; KTG: kinesiotape group; NES: neuromuscular electrical stimulation; NDT: neurodevelopmental treatment; MACS: manual ability classification system; UCP: unilateral cerebral palsy; WW: wrist wheel; WWG: wrist wheel group; PT: physiotherapy; SHUEE: Shriners Hospital upper-extremity evaluation.

**Table 2:** Methodological quality assessment using the PEDro scale.

Studies	1	2	3	4	5	6	7	8	9	10	Total Score
1. Keklicek et al., 2015 [29]	Y	N	Y	N	N	N	Y	N	Y	Y	5/10
2. Rastii et al., 2017 [28]	Y	-	-	-	-	-	Y	Y	Y	Y	5/10
3. García-Hernández et al., 2019 [30]	Y	Y	Y	-	-	-	Y	-	Y	Y	6/10
4. Acikbas et al., 2020 [31]	Y	Y	-	-	-	-	Y	Y	Y	Y	6/10
5. El-Karim et al., 2022 [32]	Y	Y	Y	-	-	-	Y	Y	Y	Y	7/10

Y: Yes; N: No. 1. random allocation; 2. concealed allocation; 3. baseline comparability; 4. blinding of individuals; 5. blinding of therapists; 6. blinding of assessors; 7. adequate follow-up; 8. intention-to-treat analysis; 9. between-group comparisons; 10. point estimates and variability.

## Data Availability

The data presented in this study are available upon request from the corresponding author. The data are not publicly available due to restrictions, e.g., privacy or ethical.

## Supplementary Materials

The following supporting information can be downloaded at: https://www.mdpi.com/article/10.3390/children11040480/s1, PRISMA 2020 Checklist. Reference [52] are cited in the Supplementary Materials.
